# Confirmation of the genetic association of *CTLA4 *and *PTPN22 *with ANCA-associated vasculitis

**DOI:** 10.1186/1471-2350-10-121

**Published:** 2009-12-01

**Authors:** Edward J Carr, Heather A Niederer, Julie Williams, Lorraine Harper, Richard A Watts, Paul A Lyons, Kenneth GC Smith

**Affiliations:** 1 Cambridge Institute for Medical Research, University of Cambridge School of Clinical Medicine, Addenbrooke's Hospital, Hills Road, Cambridge CB2 0XY, UK; 2 Department of Medicine, University of Cambridge School of Clinical Medicine, Addenbrooke's Hospital, Hills Road, Cambridge CB2 0XY, UK; 3 Division of Infection and Immunity, Medical School, University of Birmingham, Birmingham B15 2TT, UK; 4 School of Medicine, Health Policy and Practice, University of East Anglia, Norwich NR4 7TJ, UK

## Abstract

**Background:**

The genetic contribution to the aetiology of anti-neutrophil cytoplasmic antibody (ANCA)-associated vasculitis (AAV) is not well defined. Across different autoimmune diseases some genes with immunomodulatory roles, such as *PTPN22*, are frequently associated with multiple diseases, whereas specific HLA associations, such as *HLA-B27*, tend to be disease restricted. We studied ten candidate loci on the basis of their immunoregulatory role and prior associations with type 1 diabetes (T1D). These included *PTPN22*, *CTLA4 *and *CD226*, which have previously been associated with AAV.

**Methods:**

We genotyped the following 11 SNPs, from 10 loci, in 641 AAV patients using TaqMan genotyping: rs2476601 in *PTPN22*, rs1990760 in *IFIH1*, rs3087243 in *CTLA4*, rs2069763 in *IL2*, rs10877012 in *CYP27B1*, rs2292239 in *ERBB3*, rs3184504 in *SH2B3*, rs12708716 in *CLEC16A*, rs1893217 and rs478582 in *PTPN2 *and rs763361 in *CD226*. Where possible, we performed a meta-analysis with previous analyses.

**Results:**

Both *CTLA4 *rs3087243 and *PTPN22 *rs2476601 showed association with AAV, *P *= 6.4 × 10^-3 ^and *P *= 1.4 × 10^-4 ^respectively. The minor allele (A) of *CTLA4 *rs3087243 is protective (odds ratio = 0.84), whereas the minor allele (A) of *PTPN22 *rs2476601 confers susceptibility (odds ratio = 1.40). These results confirmed previously described associations with AAV. After meta-analysis, the *PTPN22 *rs2476601 association was further strengthened (combined *P *= 4.2 × 10^-7^, odds ratio of 1.48 for the A allele). The other 9 SNPs, including rs763361 in *CD226*, showed no association with AAV.

**Conclusion:**

Our study of T1D associated SNPs in AAV has confirmed *CTLA4 *and *PTPN22 *as susceptibility loci in AAV. These genes encode two key regulators of the immune response and are associated with many autoimmune diseases, including T1D, autoimmune thyroid disease, celiac disease, rheumatoid arthritis, and now AAV.

## Background

Anti-neutrophil cytoplasmic antibody (ANCA)-associated vasculitis (AAV) is characterised by small vessel inflammation and necrosis, and autoantibodies against specific neutrophil components (ANCA). The anatomical context of the inflamed vessels determines the signs and symptoms of disease. Renal and lung manifestations are common but any organ or system can be affected. AAV includes the clinical syndromes Wegener's granulomatosis (WG), microscopic polyangiitis (MPA) and Churg-Strauss Syndrome (CSS). AAV is a complex disease with both genetic and environmental factors involved in pathogenesis [1]. The magnitude of the increased familial risk is moderate; it is lower than that seen in systemic lupus erythematosus (SLE) or multiple sclerosis, but similar to that observed in rheumatoid arthritis [2]. The genes responsible for most of this risk are unknown [3]. The only consistent HLA association is with DPB1*0401 [4], however many HLA class I and class II molecules have been associated with disease in small non-replicated studies [3].

There is increasing evidence that susceptibility loci are shared between autoimmune diseases [5]. Therefore, we tested ten candidate loci on the basis of prior replicated associations with T1D [6,7]. The candidate loci tested were *PTPN22*, *IFIH1*, *CTLA4*, *IL2*, *CYP27B1*, *ERBB3*, *SH2B3*, *CLEC16A*, *PTPN2 *and *CD226*. We have previously reported an association between *IL2RA *and AAV [8]. Prior evidence supporting association exists for *CTLA4*, *PTPN22 *and *CD226 *[9-12]. CTLA-4 protein expression on CD4 T cells is increased in WG [13]. Several studies tested *CTLA4 *for association with WG or AAV. The results of these studies are conflicting. Giscombe *et al. *found an association with a SNP at position -318 (rs5742909) using 32 WG patients and 122 controls [9]. Zhou *et al. *found an association between WG and shorter (AT)n microsatellite length in the 3'UTR of *CTLA4 *in a cohort of 117 WG patients and 123 controls [14]. Slot *et al. *reported an association with a different SNP at position +49 using 102 AAV patients and 192 controls, and no effect at position -318 or the (AT)n microsatellite [10]. Finally, Spriewald *et al. *reported no association with either of the SNPs -318 or +49, or the (AT)n microsatellite in the 3'UTR of *CTLA4*, using 32 WG patients and 91 controls [15]. The prior *PTPN22 *report used 199 WG cases and 399 healthy controls and rs2476601 [11]. The *CD226 *report used 642 German WG patients and 1226 controls, but, in a parallel analysis, did not find an association in a cohort of 105 UK WG patients [12]. We sought to confirm these prior associations, and test the other T1D susceptibility loci, using a collection of 641 AAV cases and 9115 controls.

## Methods

### Patients and controls

The AAV cohort comprises subjects from four sources, all meeting the Chapel Hill diagnostic criteria [16]:

1. The MRC/Kidney Research UK (KRUK) National DNA Bank for Glomerulonephritis. Individuals were between the ages of 18 and 70 years, were ANCA seropositive, and had biopsy-proven necrotizing glomerulonephritis.

2. The UK vasculitis cohort 2 was recruited from 9 centres in the UK and comprised patients seropositive for ANCA and/or with histological evidence of small vessel vasculitis.

3. Patients recruited from the University of Birmingham. All individuals were ANCA seropositive with firm clinical and/or histological evidence of vasculitis.

4. The Lupus and Vasculitis Service, Addenbrooke's Hospital, Cambridge. All individuals were ANCA seropositive with firm clinical and/or histological evidence of vasculitis.

Genotyping was performed using TaqMan genotyping kits (Applied Biosystems) for each SNP, with fluorescence data captured using an ABI 7900 HT Fast Real-Time PCR System (Applied Biosystems) after 40 cycles of PCR.

Control genotypes for 9115 individuals from the British 1958 Birth Cohort and UK Blood Service were obtained from the Juvenile Diabetes Research Foundation/Wellcome Trust Diabetes and Inflammation Laboratory [6]. This cohort is an expansion of a dataset previously shown to be appropriate for use as UK-wide controls [17]. The 1958 Birth Cohort DNA was collected as part of an ongoing study following all births in England, Scotland and Wales in one week in 1958 http://www.b58cgene.sgul.ac.uk. This study was approved by the Cambridge Local Research Ethics Committee and by the Oversight Committee of the KRUK DNA Bank.

### Statistical analysis

Statistical analysis was performed using Prism (GraphPad) and R http://www.r-project.org. Genotype tests were performed using χ^2 ^tests for significance on 3 × 2 contingency tables. Allele analyses were performed using χ^2 ^tests for significance on 2 × 2 contingency tables. Odds ratios and 95% confidence intervals were calculated from the same 2 × 2 tables. One-sided, log-additive power calculations were performed using Quanto 1.2.4 http://hydra.usc.edu/gxe/, and the determined allele frequencies and effect sizes. Co-dominant, dominant, over-dominant, recessive and log-additive modelling was performed using the R package SNPassoc [18]. The SNPs genotyped are not within the thirteen genomic loci identified by the Wellcome Trust Case Control Consortium as showing genotype variation by geographic region of the UK [17], and it was therefore not necessary to stratify these analyses by geographical location.

## Results

In this AAV association study, eight of the ten regions tested did not show an AAV association, with *P *values between 0.19 and 0.75 (table 1). Genotypes for all SNPs did not significantly deviate from Hardy-Weinberg equilibrium in either cases or controls.

**Table 1:** Allele association testing in AAV of 11 SNPs at 10 loci

Chr	Gene	SNP	Controls n = 9115		Vasculitis cases n = 641	
			MAF	MAF	*P *value	OR (95% CI)
1p13	*PTPN22*	rs2476601 G>A (R620W)	0.10	0.13	1.4 × 10^-4^	1.40 (1.18 - 1.67)
2q24	*IFIH1*	rs1990760 T>C (A946T)	0.39	0.40	0.47	1.05 (0.93 - 1.18)
2q33	*CTLA4*	rs3087243 G>A	0.45	0.41	6.4 × 10^-3^	0.84 (0.75 - 0.95)
4q27	*IL2*	rs2069763 G>T	0.32	0.31	0.51	0.95 (0.82 - 1.10)
12q13	*CYP27B1*	rs10877012 G>T	0.33	0.32	0.34	0.93 (0.82 - 1.07)
12q13	*ERBB3*	rs2292239 A>C	0.35	0.35	0.75	0.97 (0.86 - 1.11)
12q24	*SH2B3*	rs3184504 C>T (R292W)	0.48	0.49	0.56	1.04 (0.92 - 1.17)
16p13	*CLEC16A*	rs12708716 A>G	0.35	0.36	0.24	1.08 (0.95 - 1.22)
18p11	*PTPN2*	rs1893217 T>C	0.17	0.19	0.19	1.12 (0.95 - 1.31)
18p11	*PTPN2*	rs478582 T>C	0.45	0.43	0.26	0.93 (0.83 - 1.05)
18q22	*CD226*	rs763361 C>T (G307S)	0.49	0.47	0.21	0.90 (0.77 - 1.06)

Chromosome is abbreviated to Chr; minor allele frequency is MAF; odds ratio is OR and confidence interval is CI.

Two loci, *CTLA4 *and *PTPN22*, marked by rs3087243 and rs2476601 respectively, were associated with disease (tables 1 &2). rs3087243 in *CTLA4 *was associated with disease using both genotype and allele tests (allelic *P *= 6.4 × 10^-3^; table 2). The minor allele (A) at this locus was protective, with an odds ratio (OR) of 0.84 (95% confidence interval 0.75 - 0.95). We also found both genotype and allelic associations between rs2476601 in *PTPN22 *and AAV (allelic *P *= 1.4 × 10^-4^; table 2). The minor allele (A) of rs2476601 confers susceptibility with an OR of 1.40 (95% confidence interval 1.18 - 1.67).

**Table 2:** Genotype and allele associations for *CTLA4 *rs3087243 and *PTPN22 *rs2476601 in AAV

	Controls		AAV cases		
	n	Frequency	n	Frequency	*P *value
*CTLA4 *rs3087243					
GG	2726	0.30	198	0.34	
GA	4453	0.49	282	0.49	
AA	1861	0.21	95	0.17	0.02*
					
G	9905	0.55	678	0.59	
A	8175	0.45	472	0.41	6.4 × 10^-3 ^** ^§^
*PTPN22 *rs2476601					
GG	6044	0.82	471	0.75	
GA	1298	0.18	146	0.23	
AA	70	0.01	9	0.01	5.2 × 10^-4 ^*
					
G	13386	0.90	1088	0.87	
A	1438	0.10	164	0.13	1.4 × 10^-4 ^**^§§^

* P value calculated by χ^2 ^test on 3 × 2 contingency table

** *P *value calculated by χ^2 ^test on 2 × 2 contingency table

^§ ^Odds ratio (OR) for minor allele (A) = 0.84

^§§ ^OR for minor allele = 1.40

There is a prior study reporting an association between *PTPN22 *rs2476601 and Wegener's granulomatosis [11]. The results of our meta-analysis (table 3), demonstrate a validated association for *PTPN22 *and AAV (allelic *P *= 4.2 × 10^-7^). In both *PTPN22 *studies the minor allele conferred susceptibility. The combined odds ratio for the minor allele is 1.48 (95% confidence interval 1.27 - 1.71). For a subset of our AAV patients, WG and MPA diagnoses were available. The effect of rs2476601 is present in both WG and MPA compared with the same control population (WG: n = 205, allelic *P *= 6 × 10^-11^, OR = 2.01; MPA: n = 74, allelic *P *= 4.1 × 10^-6^, OR = 2.55).

**Table 3:** Meta-analysis for *PTPN22 *rs2476601 in AAV

	Controls this study//Jagiello^1^		AAV cases this study//Jagiello^1^		
	n 7412//399	Frequency	n 626//199	Frequency	Combined *P *value
GG	6044//323	0.82//0.81	471//142	0.75//0.71	
GA	1298//72	0.18//0.18	146//52	0.23//0.26	
AA	70//4	0.01//0.01	9//5	0.01//0.03	2.2 × 10^-6 ^*
					
G	13386//718	0.90//0.90	1088//336	0.86//0.84	
A	1438//80	0.10//0.10	164//62	0.13//0.16	4.2 × 10^-7 ^** ^§^

^1 ^Data from Jagiello et al [11]

* *P *value calculated by χ^2 ^test on 3 × 2 contingency table

** *P *value calculated by χ^2 ^test on 2 × 2 contingency table

^§ ^OR for minor allele = 1.48

The prior *CTLA4 *studies with positive results used SNPs at positions -318 and +49, rather than rs3087243, precluding a meta-analysis [9,10]. We also tested for differential associations in WG or MPA but were unable to demonstrate an association with either of our smaller WG or MPA cohorts alone (data not shown).

The prior *CD226 *study reported an association with rs763361 in a cohort of German patients, but did not find an association in a collection from the University of Birmingham [12]. We also find no association between *CD226 *and AAV patients from the United Kingdom (*P *= 0.21, table 1). Excluding AAV patients from the University of Birmingham from our cohort, to minimise any potential confounding effects, does not alter the analysis (n = 391, allelic *P *= 0.38, OR = 0.93, 95% CI = 0.78 - 1.10, power = 27.3%).

For both *CTLA4 *rs3087243 and *PTPN22 *rs2476601, we tested the genotype data (table 2) using several different models of inheritance (co-dominant, dominant, over-dominant, recessive and log-additive). No single model had a clearly superior fit at either locus (table 4).

**Table 4:** Models of inheritance for the *CTLA4 *rs3087243 and *PTPN22 *rs2476601 associations in AAV

	*P *value of indicated model				
	Co-dominant	Dominant	Over-dominant	Recessive	Log-additive
*CTLA4* rs3087243	0.02	0.03	0.92	0.02	5.8 × 10^-3^
*PTPN22* rs2476601	8.5 × 10^-4^	1.8 × 10^-4^	4.3 × 10^-4^	0.26	2.0 × 10^-4^

## Discussion

*CTLA4 *and *PTPN22 *are now firmly established as AAV susceptibility loci, along with *HLA-DPB*, with associations confirmed in two separate cohorts. Clarification of the potentially complex role of the *CTLA4 *locus in AAV, as implied by apparently conflicting prior studies [9,10,14,15], would be provided by a larger, more detailed study of the locus. *PTRN3 *and *AAT *are the only other confirmed AAV associations [1].

*CTLA4 *encodes cytotoxic T lymphocyte antigen 4 (CTLA-4), a negative regulator of T cell activation. *Ctla4*^-/- ^mice die by 3-4 weeks of age, with lymphadenopathy and splenomegaly, consisting of T cell expansions expressing activation markers [19], and lymphocytic infiltration and tissue destruction of many organs, including heart and pancreas [20]. Numerous subsequent studies have revealed that CTLA-4 has complex biology; it is known to bind CD80 and CD86 with higher affinity than CD28 [21,22], it is expressed by both regulatory and effector T cells, and it is synthesised as soluble and transmembrane isoforms [23] with the majority of CTLA-4 retained intracellularly. Specific deficiency of *Ctla4*^-/- ^in natural Foxp3+CD4+ regulatory T cells results in spontaneous autoimmune disease [24]. The precise role of CTLA-4 in the maintenance of peripheral tolerance remains unclear, with no unifying model of CTLA-4 function [25]. Human polymorphisms in *CTLA4 *are associated with several autoimmune diseases, including type 1 diabetes (T1D), Graves' disease [6,26], Hashimoto's thyroiditis [27], celiac disease [28], and rheumatoid arthritis (RA) [29].

CD4 T cells from AAV patients express higher levels of membrane-bound CTLA-4 protein than CD4 T cells from healthy controls [13]. The common G allele of rs3087243, which confers susceptibility to AAV, does not alter expression of full length CTLA4 mRNA [26]. Despite the G allele of rs3087243 associating with a lowered expression of the CTLA4 transcript encoding the soluble form of the protein [26], genotype at rs3087243 does not correlate with soluble CTLA-4 protein in serum from T1D patients or from autoantibody positive or negative healthy controls [30]. A different SNP, rs5742909, in the *CTLA4 *promoter region may be associated with expression level [31] and there is some evidence of association with WG [9]. Linkage disequilibrium between rs3087243 and rs5742909 is low (r^2 ^= 0.074; CEPH dataset, http://hapmap.ncbi.nlm.nih.gov/. Thus rs57942909 may have an independent effect to that conferred by rs3087243.

In addition to the effects on the expression of *CTLA4 *isoforms, the two alleles of rs3087243 are associated with altered T cell phosphorylation levels [32]. CD4 T cells from carriers of the AAV risk-conferring G allele exhibit greater levels of phospho-LAT, phospho-LCK, phospho-ZAP70, and phospho-SLP76 after anti-CD3 treatment. There have been no reported differences in CTLA-4 protein expression on CD4 T cells between donors of all rs3087243 genotypes. The lower activation threshold of T cells from carriers of the G allele of rs3087243 might thus contribute to T cell activation and autoimmune disease susceptibility. Taken together these data suggest that genotype at rs3087243 could impact upon both the expression of *CTLA4 *mRNA isoforms and the subsequent level and function of the translated protein.

*PTPN22 *encodes a second negative regulator of T cell activation, LYP. The SNP rs2476601 is a non-synonymous change substituting arginine for tryptophan at amino acid residue 620. *PTPN22 *is being increasingly recognised as a central player in T cell regulation, being a direct target for inhibition by the regulatory T cell transcription factor Foxp3 [33] and by the T cell modulating mir-181a [34]. The predisposing allele in T1D, RA and AAV is Trp^620 ^(the A allele). T cell receptor stimulation of T cells from carriers of the Trp^620 ^allele, resulted in decreased interleukin 2 production due to increased phosphatase activity [35]. The functional implications of this SNP also extend to B cells, where the Trp^620 ^allele reduces activation thresholds in an analogous manner to T cells, perhaps allowing autoantibody production by B cells in addition to the effects of the Trp^620 ^allele in T cells [36]. However, whilst mice lacking *Pep *(the murine ortholog of *PTPN22*) have spontaneous germinal centre formation, autoantibodies have not been described [37]. While the Trp^620 ^*PTPN22 *allele is associated with susceptibility in many diseases [38], the alternative Arg^620 ^allele confers risk in others (such as Crohn's disease [39]). This underlines the importance of the pathway controlled by *PTPN22 *in autoimmunity, but indicates distinct regulatory changes in the pathway are involved in different disease states. It has been suggested that the Trp^620 ^allele is implicated in autoimmune diseases associated with autoantibody production, such as AAV or T1D [36], and indeed the Trp^620 ^allele of rs2476601 in *PTPN22 *is associated with both WG and MPA.

*CTLA4*, *PTPN22 *and *IL2RA *are all genes associated with the regulation of T cell activation, via the modulation of signalling in effector T cells and the control of regulatory T cells. All of these genes are associated with multiple autoimmune diseases, including T1D, autoimmune thyroid disease and AAV [6,8,26,40-42]. This may in part explain the familial co-segregation seen between some of these diseases, for example between T1D and autoimmune thyroid disease [26,43]. AAV however does not show clear evidence for co-segregation with other autoimmune diseases, implying the existence of susceptibility loci unique to AAV pathogenesis. These unique loci, by their nature, are difficult to identify with candidate gene studies, demonstrating the need for a genome-wide association study in AAV.

## Conclusion

We confirm two associations with AAV in *CTLA4 *and *PTPN22*, both important regulators of the immune response. There are now five confirmed AAV susceptibility loci: *HLA-DPB1*, *CTLA4*, *PTPN22*, *PRTN3 *and *AAT*.

## Competing interests

The authors declare that they have no competing interests.

## Authors' contributions

KGCS & PAL conceived and designed the study. JW, LH & RAW provided access to DNA samples. EJC & HAN performed the SNP genotyping and its analysis. The manuscript was written by EJC & KGCS. All authors read and approved the final manuscript.

## Pre-publication history

The pre-publication history for this paper can be accessed here:

http://www.biomedcentral.com/1471-2350/10/121/prepub
